# Enhanced efficacy of curcumin with phosphatidylserine-decorated nanoparticles in the treatment of hepatic fibrosis

**DOI:** 10.1080/10717544.2017.1399301

**Published:** 2017-12-07

**Authors:** Ji Wang, Wen Pan, Ying Wang, Wan Lei, Bin Feng, Caigan Du, Xiao-juan Wang

**Affiliations:** ^a^ State Key Laboratory of Military Stomatology and National Clinical Research Center for Oral Diseases and Shaanxi Engineering Research Center for Dental Materials and Advanced Manufacture, Department of Pharmacy, School of Stomatology, The Fourth Military Medical University Xi’an PR China; ^b^ Department of Urologic Sciences, University of British Columbia, Jack Bell Research Centre Vancouver BC Canada

**Keywords:** Phosphatidylserine, curcumin, nanostructured lipid carriers, liver fibrosis, macrophages

## Abstract

Hepatic macrophages have been considered as a therapeutic target for liver fibrosis treatment, and phosphatidylserine (PS)-containing nanoparticles are commonly used to mimic apoptotic cells that can specifically regulate macrophage functions, resulting in anti-inflammatory effects. This study was designed to test the efficacy of PS-modified nanostructured lipid carriers (mNLCs) containing curcumin (Cur) (Cur-mNLCs) in the treatment of liver fibrosis in a rat model. Carbon tetrachloride-induced liver fibrosis in rats was used as an experimental model, and the severity of the disease was examined by both biochemical and histological methods. Here, we showed that mNLCs were spherical nanoparticles with decreased negative zeta potentials due to PS decoration, and significantly increased both mean residence time and area under the curve of Cur. In the rats with liver fibrosis, PS-modification of NLCs enhanced the nanoparticles targeting to the diseased liver, which was evidenced by their highest accumulation in the liver. As compared to all the controls, Cur-mNLCs were significantly more effective at reducing the liver damage and fibrosis, which were indicated by in Cur-mNLCs-treated rats the least increase in liver enzymes and pro-inflammatory cytokines in the circulation, along with the least increase in collagen fibers and alpha smooth muscle actin and the most increased hepatocyte growth factors (HGF) and matrix metalloprotease (MMP) two in the livers. In conclusion, PS-modified NLCs nanoparticles prolonged the retention time of Cur, and enhanced its bioavailability and delivery efficiency to the livers, resulting in reduced liver fibrosis and up-regulating hepatic expression of HGF and MMP-2.

## Introduction

Liver fibrosis is characterized by excessive extracellular matrix (ECM) deposition and severe structural and functional alterations in the liver (Tacke & Zimmermann, 2014). Hepatic macrophages, accounting for approximately 80–90% of all macrophages in the body, are involved in maintaining tissue homeostasis in the liver as well as act as either pro- or antifibrotic factor in the pathogenesis of liver fibrosis (Bartneck et al., 2014). These macrophages secrete profibrotic mediators such as transforming growth factor-β1 (TGF-β1) and platelet-derived growth factor that directly activate fibroblasts (Ju & Tacke, 2016). On the other hand, they produce matrix metalloproteases (MMPs) and release cytokines to promote inflammation and fibrosis resolution (Pellicoro et al., 2012). They also remove dead cells including apoptotic cells and debris by phagocytosis to suppress proinflammatory and profibrotic signals (Wynn & Barron, 2010). Thus, hepatic macrophages are a potential therapeutic target in the treatment of liver fibrosis.

The potential of using nanoparticles for the treatment of liver diseases has been recently examined. The nanoparticles can accumulate in the liver and are subsequently internalized by hepatic macrophages, which make them ideal candidates for treating liver diseases because they are expected to deliver potent anti-fibrotic drugs specifically to hepatic macrophages (He et al., 2013). Many researchers have reported that nanoparticles are promising for therapeutic targeting hepatic macrophages. For examples, dexamethasone-loaded liposomes significantly reduce liver injury and liver fibrosis in inflammatory liver diseases (Bartneck et al., 2014), a polymeric nanoparticle formulation of curcumin (Cur) has been reported to markedly attenuate carbon tetrachloride (CCl_4_)-induced liver fibrosis by inhibiting production of pro-inflammatory cytokines (Bisht et al., 2011), and mannose-modified trimethyl chitosan-cysteine conjugate nanoparticles are highly effective polymeric vehicles for oral delivery of TNF-α siRNA to macrophages, which then protect mice from inflammation-induced liver damage (He et al., 2013).

Phosphatidylserine (PS) is an anionic phospholipid component of cell membranes, and it normally presents in the inner cell membrane of the phospholipid bilayer (Bagalkot et al., 2016). When a cell undergoes apoptosis, a significant amount of PS is transferred to the outer cell membrane. The exposed PS acts as a specific recognition signal for phagocytosis of apoptotic cells by macrophages (Ravichandran, 2011). Nanomaterials containing synthetic PS have been developed to mimic apoptotic cells either to exhibit immune-regulatory functions in different models of inflammation (Harel-Adar et al., 2011), or to deliver agents to specific sites enriched with macrophages (Kansal et al., 2012; Ogawa et al., 2014; Ogawa et al., 2015). Our previous work has demonstrated that Cur-modified nanostructured lipid carriers (mNLCs) has superior cellular drug delivery properties and anti-inflammatory effects, which may potentially be used in the treatment of macrophages-mediated diseases (Wang et al., 2016). Besides, it has been reported that administration of liposomes to chronically injured mice activates phagocytic activity of macrophages, similar to the effects of phagocytosis of cellular debris, which further enhances the restorative macrophage phenotype *in vivo*, and accelerates fibrosis reduction (Ramachandran et al., 2012). Other investigations also reveal that one-time apoptotic cell instillation prevents lung inflammation and fibrosis (Yoon et al., 2015), and this effect is due to apoptotic cells clearance and couples engulfment to production of anti-inflammatory cytokines and suppression of inflammatory pathways (Adhyatmika et al., 2015).

Based on the above findings, we hypothesized that Cur-mNLCs might have potential for the treatment of liver fibrosis. It has been reported that Cur exerts potent anti-fibrotic efficacies (Fu et al., 2007; Tu et al., 2012b; Yao et al., 2013). Tu et al. have demonstrated that Cur could attenuate inflammation and fibrosis in mouse models of acute and chronic liver injury (Tu et al., 2012a, 2012b). Thus, it is anticipated that Cur would be specifically delivered to macrophage-enriched liver with the aid of PS, resulting in attenuating liver fibrosis.

**Figure 1.**
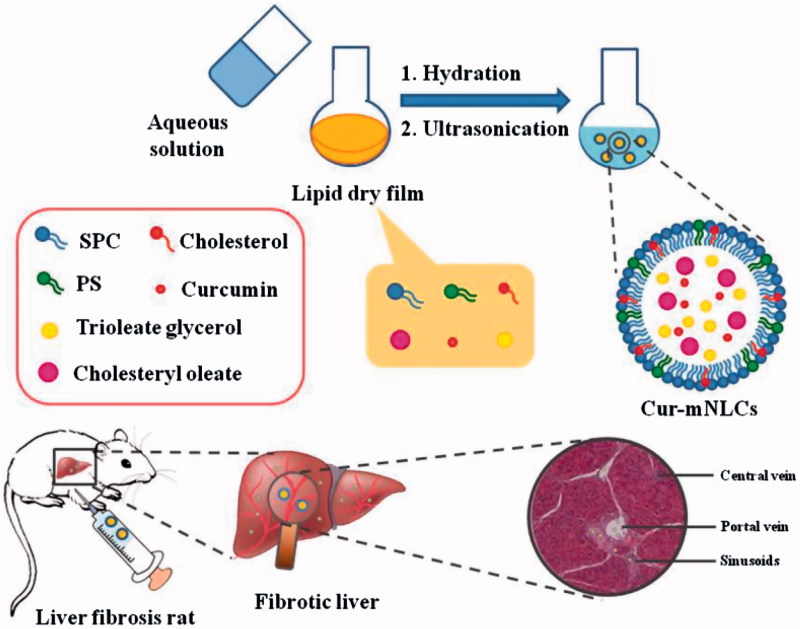
A schematic diagram of preparation procedures and liver targeting properties of Cur-mNLCs.

In this study, Cur-mNLCs were prepared by using thin-film dispersion method, and their particle size, zeta potential, morphology, entrapment efficiency (EE) and drug-loading (DL) capacity were evaluated. The pharmacokinetic behaviors and liver targeting properties of Cur-mNLCs were investigated in rats with liver fibrosis. Finally, the efficacy of Cur-mNLCs against fibrosis regression was examined in a rat model. The main scheme of our study was presented in Figure 1.

## Materials and methods

### Chemical reagents and animals

Curcumin (denoted as Cur, purity of 98%) was purchased from Aladdin Industrial Incorporation (Ontario, CA); soybean phosphatidylcholine (Lipoid S 100) from Lipoid GmbH (Ludwigshafen, Germany), PS (1,2-diacyl-sn-glycero-3-phospho-L-serine) and cholesterol from Sigma-Aldrich (St. Louis, MO), cholesteryl oleate from Alfa Aescar/Thermo Fisher Scientific (Lancashire, UK), Colchicine from (Xishuangbanna Pharmaceutical Co., Ltd) and trioleate glycerol from Tokyo Chemical Industry Co., Ltd. (Tokyo, Japan). Near infrared fluorescent dye DiR was from AAT Bioquest (Sunnyvale, CA). Deionized water (18.2 MΩ) was collected from an analytic ultra-pure water system (ELGA, High Wycombe, UK).

Male Sprague–Dawley rats (7–9 weeks old; body weight: 200 ± 20 g) were purchased from the Experimental Animal Research Center of the Fourth Military Medical University (Xi’an, Shaanxi, China), and were maintained at 12 h light/12 h dark cycles with free access to food and water. Whole blood samples were collected from the orbit. All the animal use procedures were approved and supervised by the Animal Care and Use Committee of the Fourth Military Medical University according to the Chinese Council on Animal Care guidelines.

### Preparation and characterization of different NLCs

Two different Cur-containing NLCs, 0% PS (denoted as Cur-NLCs) and 8% PS (denoted as Cur-mNLCs), were prepared by using the thin-film dispersion method as previously reported (Wang et al., 2016). For blank PS-containing NLCs (denoted as B-mNLCs), the preparation procedure was the same as for Cur-mNLCs except that Cur was omitted.

The morphology and structure of the resultant NLCs were examined by using transmission electron microscopy (TEM, Tecnai G2, FEI, Hillsboro, TX). The average size and zeta potentials of NLCs were determined by using a dynamic light scattering analyzer (Nano-ZS90, Malvern, UK). Encapsulating efficiency (EE) and DL of Cur-NLCs and Cur-mNLCs were measured as previously reported (Wang et al., 2016).

### Animal model of liver fibrosis

Male Sprague–Dawley rats were randomly divided into two groups: Sham control (total 9 rats) and disease group (total 97 rats). The sham control group was intraperitoneally injected olive oil twice weekly, whereas the disease group was given intraperitoneally injection of CCl_4_/olive oil (1:1, v/v) twice weekly at a dose of 2 ml/kg body weight. At the end of eight weeks of injection, three rats were randomly selected from each group, and the pathological examination of their liver tissue sections after hematoxylin and eosin (H&E) and Sirus Red staining was performed. When compared with sham control animals (absence of any pathological change), all the liver sections from disease group indicated the typical fibrotic pathological features (data not shown), which confirmed the presence of liver fibrosis in rats from the disease group.

### Pharmacokinetics of Cur in different carriers in rats with liver fibrosis

Eighteen rats with liver fibrosis (in the disease group) were selected for pharmacokinetics studies and randomly divided into three groups (*n* = 6): (1) Free-Cur (Cur dissolved in 20% Tween 80 at a concentration of 2 mg/mL); (2) Cur-NLCs; (3) Cur-mNLCs, respectively. All the solutions were administered by intraperitoneal injection at Cur dose of 15 mg/kg. The blood samples (1 mL) were collected from orbit at different time points, followed by centrifugation at 3000 rpm for 10 min. The supernatants or plasma samples were stored at −20 °C until they were analyzed.

Rat plasma (200 µL) were mixed with 2 µL of emodin (an internal standard), followed by extraction with 400 µL of methanol, vortexed for 1 min and centrifuged at 13,000 rpm for 10 min. The upper organic layer was transferred to a 1.5 mL tube and dried under nitrogen. The residuals were re-dissolved in 200 µL of methanol, vortexed for 3 min, and centrifuged at 13,000 rpm for 10 min again. The final supernatants (5 µL) were used for Cur content analysis by using LC-MS/MS as described below. The pharmacokinetics parameters were calculated by using a non-compartmental model (WinNonlin software version 5.2.1, Pharsight Corporation, Mountain View, CA).

### LC-MS/MS conditions

The ultra-high performance liquid chromatography (UHPLC) (Agilent 1290 Infinity; Agilent Technologies, CA) was connected to a mass spectrometer with an electrospray ionization source (6460 LC-MS/MS triple quadrupole system, Agilent Technologies, CA), and the system was run by negative ionization mode. Wonda Cract ODS-2 (150 mm × 4.6 mm, 5 µm) was used for chromatographic separation. The column temperature was maintained at 25 °C. The mobile phase consisted of acetonitrile and water (90:10, v/v). The flow rate was 0.3 ml/min and the injection volume was 5 µL.

The MS operational parameters were as follows: capillary voltage at 3.5 kV, nebulizer at 45 psi, drying and sheath gas temperature was both set at 350 °C. Nitrogen was used as the drying and sheath gas with a flow rate of 6 and 11 L/min, respectively. Quantification was performed using multiple-reaction monitoring method with the transitions of *m/z* 367.0 → 217.0 for Cur, *m/z* 269.0 → 225.1 for emodin, respectively. Fragmentor voltage and collision energy was set at 110 V and 0 eV for curcumin, 137 V and 29 eV for emodin, respectively. MassHunter (Version B.04.10) was used for the system control, data processing and data acquisition for both LC and mass spectrometry.

### Determination of nanoparticle targeting properties in rats with liver fibrosis

#### *Ex vivo* imaging

Near-infrared fluorescent dye DiR-loaded NLCs without PS (DiR-NLCs) or with PS (DiR-mNLCs) were prepared in the same way as Cur-loaded NLCs, except that Cur was replaced by DiR.

Both DiR-NLCs and DiR-mNLCs (0.2 mg/kg) were injected intraperitoneally into the rats with liver fibrosis (total four rats), respectively. At 2 or 4 h (one rat at each time point) after injection, the animals were sacrificed, and major organs (hearts, livers, spleens, lungs, kidneys, and brains) from each animal were harvested. After appropriate disposal, the *ex vivo* imaging of each organ was performed and viewed by using an IVIS Lumina II Imaging System (Caliper Life Sciences, MA) equipped with an excitation band pass filter at 748 nm and an emission at 780 nm. Images were analyzed using Living Imaging software (Version 4.2, Caliper Life Sciences).

#### Tissue distribution in rats with liver fibrosis

Thirty-six rats with liver fibrosis were selected and randomly divided into three groups (*n* = 12): (1) Free-Cur, (2) Cur-NLCs, and (3) Cur-mNLCs, respectively. All the solutions were intraperitoneally injected at a dose of 15 mg/kg of Cur. Animals (six rats at each time point) were euthanized at 2 or 4 h after injection, and the blood and major organs (heart, liver, spleen, lung, kidney, and brain) were collected, respectively. All the tissue samples were rinsed in saline solution, air-dried and weighed. The organ was cut into small pieces and thoroughly homogenized in the saline to obtain 500 mg tissue/ml homogenate. Finally, the concentrations of Cur in both plasma and different tissues were measured by using LC-MS/MS as described above.

### Determination of anti-fibrosis activity in rats with liver fibrosis

Forty-two rats (six from the sham group; 36 from the disease group) were randomly divided into seven groups (*n* = 6): (1) Naive group: only received intraperitoneal injections of olive oil twice weekly; (2) Vehicle group: rats from the disease group without any drug treatment; (3) Free-Cur group: rats from the disease group treated with Free-Cur solution (15 mg/kg Cur, dissolved in 20% Tween 80, intraperitoneal injection every other day); (4) Cur-NLCs group: rats from the disease group treated with Cur-NLCs (15 mg/kg Cur, intraperitoneal injection every other day); (5) Cur-mNLCs group: rats from the disease group treated with Cur-mNLCs (15 mg/kg Cur, intraperitoneal injection every other day); (6) B-mNLCs group: rats from the disease group treated with B-mNLCs (same volume and treatment as Cur-mNLCs); finally, (7) Colchicine group: rats from the disease group treated with colchicine (0.1 mg/kg) once a day by intragastric administration. Colchicine has been used in the treatment of chronic liver disease or fibrosis (Muntoni et al., 2010; Xi et al., 2016) and was included in this study as a reference group. At the end of eight weeks of the treatment, the rats were sacrificed, and the serum and organs were recovered.

#### Blood biochemical assay

Serum was collected and analyzed for its alanine aminotransferase (ALT), aspartate aminotransferase (AST), alkaline phosphatase (AKP) and total bilirubin (T-Bil). These parameters were measured by using commercial kits according to the manufacturer’s instructions (Nanjing Jiancheng Biotech, Nanjing, China).

#### Liver histology and Sirius Red staining for fibrosis

Liver tissues were recovered and immediately fixed in 4% paraformaldehyde, embedded in paraffin, then sectioned into 4-µm sections. The tissue sections were finally stained with H&E or Sirius Red following the routing protocols in the pathology laboratory. Sirius Red stained pictures were analyzed by integrated optical density (IOD) quantification (Image-Pro Plus 6.0, IPP 6.0) in a blinded fashion.

#### Immunohistochemistry for collagen I and alpha smooth muscle actin (α-SMA)

The liver tissue sections were deparaffinized, rehydrated, and subjected to heat-induced antigen retrieval. Sections were blocked and incubated with rabbit polyclonal anti-collagen I (1:200; Abcam, UK) or rabbit polyclonal anti-α-SMA (1:75; Abcam, UK) overnight at 4 °C. Then sections were washed and incubated with HRP-conjugated goat anti-rabbit IgG secondary antibodies. Finally, the sections were incubated with 3,3′-diaminobenzidine tetrachloride, and visualized by light microscopy.

After images were obtained, the quantification of the positively stained areas expressed as IOD was performed using IPP 6.0 software.

#### Immunofluorescence analysis of hepatocyte growth factor (HGF)

Tissue specimens of the liver were immediately embedded in OCT medium after harvest and then cryogenically sectioned. The sections were blocked with 10% normal goat serum (Sigma-Aldrich) and 0.1% Triton X-100 in PBS, and then incubated with primary antibodies (1:50; rabbit polyclonal anti-HGF; Abcam, UK) overnight at 4 °C. After three washes with PBS, the sections were incubated with Alexa Fluor 488-labeled goat anti-rabbit IgG (H + L) secondary antibodies (1:100) at 37 °C for 2 h. The sections incubated with secondary antibodies alone were used as a negative control. Finally, nucleus was stained with 4′,6-diamidino-2-phenylindole. The staining density was visualized under CLSM (FluoView™ FV1000, Leica Microsystems, Wetzlar, Germany).

#### Measurement for proinflammatory cytokines

Enzyme linked immunosorbent assay (ELISA) kits (Westang Biotechnology Co., Ltd, Shanghai, China) were used to determine the concentrations of TNF-α, IL-1β, and IL-6 in serum according to the manufacturer’s instructions.

#### Gelatin zymography analysis for MMP-2

Gelatinase activity of MMP-2 was determined by gelatin zymography. In brief, liver samples were homogenized with NP40 (1:30 mass ratio) on ice. The homogenate was centrifuged at 13,000 g for 10 min, and then the BCA method was used to determine protein content in the supernatant. Dilute samples at 1:1 with 2 × SDS-PAGE non-reducing buffer. Equal amounts of protein were loaded into 9% SDS-PAGE containing 10% gelatin to separate proteins. After electrophoresis at 40 mA, gels were washed three times with 2.5% Triton X-100 for 30 min and then incubated in a solution containing 50 mM Tris and 10 mM CaCl_2_, pH 7.5 at 37 °C for 18 h, and then were stained with 0.25% Coomassie blue for 15 min, followed by being decolored with a solution containing 7% acetic acid and 5% methanol. The intensity of bands was quantified by densitometry analysis using Gel-Pro Analyzer (Media Cybernetics, Inc., MD).

### Statistical analysis

Data were presented as mean ± standard derivation (SD) of each group. The difference between two groups was analyzed by using Student’s *t*-test with two-tailed distribution, and comparisons of parameters among multiple groups were made with a one-way ANOVA. A *p* value of < .05 was considered significant.

## Results

### PS loading changed some characters of NLCs

The shape of all three different types of particles (Cur-NLCs, Cur-mNLCs, and B-mNLCs) was examined by using TEM, and they all exhibited similar spherical shape in TEM images (Figure S1). Their size, polydispersity index (PDI), zeta potential, EE and DL were examined, and the data were listed in Table 1. The mean particle diameters of all these NLCs were in the range of nano-scale. PS increased zeta potentials of Cur-mNLCs and B-mNLCs as compared to Cur-NLCs (Cur-NLCs vs. Cur-mNLCs or B-mNLCs, *p < .*01). While both EE and DL of Cur-mNLCs were not significantly changed by PS decoration.

**Table 1.** Particle size, PDI, zeta potential, EE, and DL of different NLCs particles.

Samples	Particle size (nm)	PDI	zeta potential (mV)	EE (%)	DL (%)
Cur-NLCs	216.3 ± 1.54	0.033 ± 0.004	−2.08 ± 0.32	86.72 ± 0.22	3.95 ± 0.07
Cur-mNLCs	204.6 ± 1.97	0.067 ± 0.002	−46.29 ± 0.48*	89.06 ± 0.47	4.19 ± 0.08
B-mNLCs	208.3 ± 1.26	0.084 ± 0.003	−44.57 ± 0.25*	–	–

Data were presented as mean ± standard derivation (SD) of three separate determinants (*n* = 3).

* *p < .*01, compared with Cur-NLCs.

### PS-enhanced the retention times of Cur encapsulated in NLCs in sera

**Figure 2.**
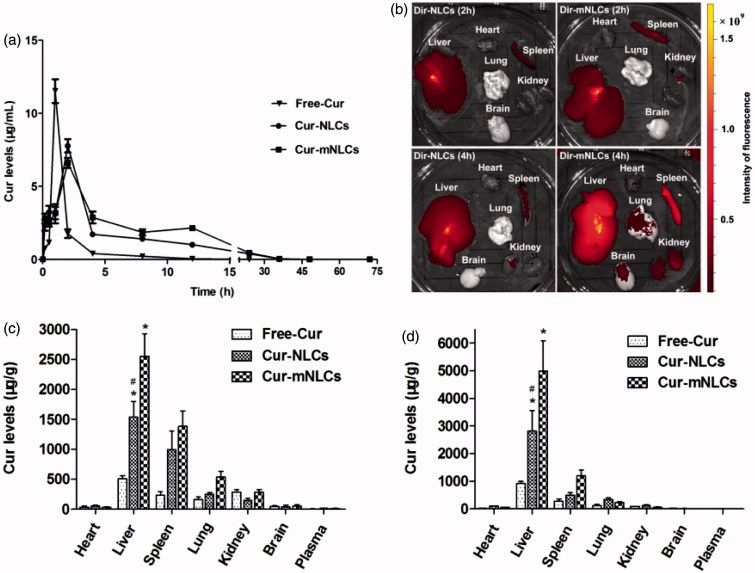
PS enhanced the retention times of Cur encapsulated in NLCs in sera as well as Cur delivery by NLCs to the liver *in vivo*. (a) The changes of plasma concentration of Cur after i.p. injection of Free-Cur solution, Cur-NLCs, and Cur-mNLCs in rats with liver fibrosis. Data in each point were presented as mean ± SD (*n* = 6). (b) *Ex vivo* imaging of organs in rats with liver fibrosis indicated that PS decoration improved liver targeting properties of nanocarriers. Data were presented as a typical image of major organs from one rat at each time point. (c–d) Tissue distribution of Cur after i.p. injection of Free-Cur solution, Cur-NLCs and Cur-mNLCs in rats with liver fibrosis at 2 h (c) and 4 h (d). PS decoration enhanced NLCs liver targeting properties. Data were presented as mean ± SD at each point (*n* = 6). *p < .01, compared with Free-Cur; ^#^ *p < *.01, compared with Cur-mNLCs.

The pharmacokinetics of Cur in different nanoparticle carriers (Cur-NLCs and Cur-mNLCs compared to Free-Cur solution) was examined in rats with liver fibrosis, and the Cur plasma levels over the time after i.p. administration were determined by using UHPLC-MS/MS method. As seen in Figure 2(a), administration of Cur-NLCs and Cur-mNLCs significantly altered pharmacokinetic behaviors of Cur as compared to that of Free-Cur solution. The Cur from the Free-Cur solution was quickly diffused into the systemic circulation (*t*_max_ = 1 h), while the sustained Cur release from Cur-NLCs or Cur-mNLCs was significantly prolonged (*t*_max_=2 h). As compared with the Free-Cur solution (Table 2), both Cur-NLCs and Cur-mNLCs similarly decreased *C*_max_ of Cur (Free-Cur vs. Cur-NLCs or Cur-mNLCs, *p < .*01; Cur-NLCs vs. Cur-mNLCs, *p* = .173), or increased its MRT (Free-Cur vs. Cur-NLCs or Cur-mNLCs, *p < .*01; Cur-NLCs vs. Cur-mNLCs, *p* = .052). In the analysis of AUC of Cur, again both Cur-NLCs and Cur-mNLCs increased the AUC (Free-Cur vs. Cur-NLCs or Cur-mNLCs, *p < .*01), and PS modification (Cur-mNLCs) further significantly enhanced it (Cur-NLCs vs. Cur-mNLCs, *p* = .002). These data indicated that the encapsulation of Cur in the NLCs prolonged the retention time *in vivo* and enhanced its bioavailability, which was further enhanced by the PS modification.

**Table 2.** Pharmacokinetic parameters of Cur in different carriers after i.p. administration.

Formulations	*t*_max_ (h)	*C*_max_ (μg/mL)	MRT_last_ (h)	MRT_INF_obs_ (h)	AUC_last_ (μg/mL*h)	AUC_INF_obs_ (μg/mL*h)	V_z_F_obs_ (L/kg)	Cl__F_obs_ (L/h/kg)
Free-Cur	1	11.520 ± 2.005	2.472 ± 0.316	2.574 ± 0.380	12.344 ± 2.024	12.376 ± 2.040	8.644 ± 2.698	1.244 ± 0.238
Cur-NLCs	2	7.779 ± 1.103*	8.289 ± 0.707*	8.312 ± 0.708*	36.968 ± 4.934*^,^^§^	36.986 ± 4.932*^,^^§^	2.293 ± 0.634*	0.411 ± 0.052*
Cur-mNLCs	2	6.618 ± 0.835*	9.151 ± 0.928*	9.177 ± 0.932*	48.604 ± 7.948*	48.631 ± 7.944*	1.913 ± 0.452*	0.315 ± 0.051*
Free-Cur vs. Cur-NLCs		<0.01	<0.01	<0.01	<0.01	<0.01	<0.01	<0.01
Free-Cur vs. Cur-mNLCs		<0.01	<0.01	<0.01	<0.01	<0.01	<0.01	<0.01
Cur-NLCs vs. Cur-mNLCs		0.173	0.049	0.052	0.002	0.002	0.691	0.266

Data were presented as mean ± SD (*n* = 6). *t*_max_: the time to achieve the maximum concentration; *C*_max_: the maximum concentration in serum; MRT_last_: the mean residence time to last sampling time; MRT_INF_obs_: the mean residence time to infinity; AUC_last_: the concentration-time curve to the last sampling time; AUC_INF_obs_: the concentration-time curve to infinity; V_z_F_obs_: apparent volume of distribution; Cl__F_obs_: plasma clearance.

*p < .01, compared with Free-Cur; #*p* < .05, compared with Cur-mNLCs; §*p* < .01, compared with Cur-mNLCs.

### PS-enhanced the Cur delivery by NLCs to the liver *in vivo*

Liver targeting properties of different NLCs were tested by using *ex vivo* imaging first. After injection of DiR-NLCs and DiR-mNLCs in the rats, the fluorescence in each organ was examined at both 2 and 4 h. As seen in Figure 2(b), both nanoparticles were mainly taken up by the liver and the spleen, and were clearly seen at 2 h (Figure 2(b), upper panel). At 4 h, other organs (the kidneys, lung, and brain) also were seen red in DiR-mNLCs group (Figure 2(b), lower panel). Compared to the intensity of the color in DiR-NLCs-targeted liver, DiR-mNLCs-targeted livers exhibited much stronger fluorescence, indicating the superior liver targeting properties of DiR-mNLCs.

The PS-enhanced liver targeting of NLCs was further confirmed by Cur tissue distribution delivered by different NLCs in rats with liver fibrosis. Figure 2(c,d) showed that at both 2 and 4 h after injection, the highest level of Cur in all three groups (Free-Cur, Cur-NLCs, and Cur-mNLCs) was found in the liver, and the second in the spleen, which were consistent with the imaging data in Figure 2(b). Furthermore, the Cur levels in the liver were increased three and five-fold by Cur-NLCs and Cur-mNLCs as compared to Free-Cur solution (Free-Cur vs. Cur-NLCs or Cur-mNLCs, *p < .*01), respectively, and Cur accumulation in the liver was further significantly enhanced by PS modification of NLCs nanoparticles (Cur-mNLCs vs. Cur-NLCs, *p < .*01). It was also noticed that at 4 h post-injection, the Cur levels in all these three groups were decreased in all the tissues except of the livers, suggesting that Cur from these three different delivery systems may be specifically accumulated in the liver. Taken together, both *ex vivo* imaging and tissue distribution studies indicated that PS decoration enhanced the liver targeting properties of a drug delivered by NLCs nanoparticles.

### Treatment with Cur-mNLCs significantly reduced CCl_4_-induced elevation of liver disorder markers and pro-inflammatory cytokines in sera

As shown in Figure S2a, the liver disorder marker ALT, AST and AKP, and T-Bil in the sera were significantly elevated in rats following CCl_4_-induced liver injury (Naive vs. Vehicle, *p < .*01). When these rats were treated with different Cur formulations (Free-Cur, Cur-NLCs, and Cur-mNLCs), blank nanoparticles (B-mNLCs) and a reference drug colchicine, all the treatments including B-mNLCs significantly reduced the serum levels of these liver disorder markers as compared to vehicle-treated group (*p < .*05). Among these different treatments, Cur-mNLCs exhibited the most significant effect on the reduction of serum ALT, AST, and AKP, which was indicated by no statistical difference between Naive and Cur-mNLCs-treated group in ALT (*p* = .786), AST (*p* = .693) and AKP (*p* = .524), whereas in other treatment groups the levels of these markers were higher than those in naive rats (Figure S2a). Also, it was noticed that the difference between Cur-NLCs and Cur-mNLCs was significant (ALT: *p* = .014; AST: *p* = .004), suggested that PS-modification of NLCs enhanced Cur efficacy in reducing serum levels of ALT and AST.

The serum levels of proinflammatory cytokines (TNF-α, IL-1β, and IL-6) were also determined by using ELISA kits. Similar to the liver disorder markers, all of these pro-inflammatory cytokines were remarkably up-regulated in non-drug treated CCl_4_-treated rats (*p < .*01, compared to Naive group) (Figure S2b), and all the drug treatments lowered their levels (*p < .*01). Again, rats receiving Cur-mNLCs had the lowest mean level of these cytokines compared to other treatment groups (Figure S2b).

### Treatment with Cur-mNLCs significantly reducedCCl_4_-induced liver fibrosis

**Figure 3.**
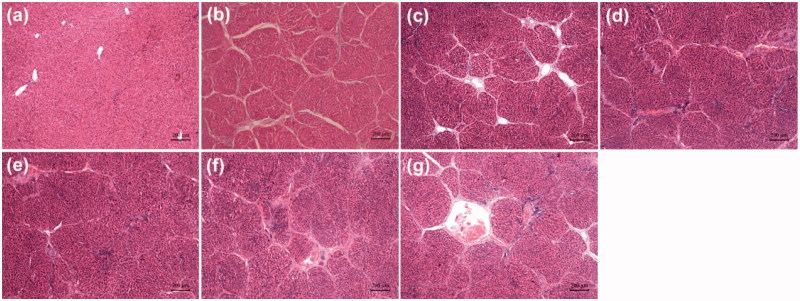
Effects of different drug formulations on the histological changes in the liver of CCl_4_-treated rats as shown by H&E staining. (a) Naive group, (b) Vehicle group (positive control), (c) Free-Cur group, (d) Cur-NLCs group, (e) Cur-mNLCs group, (f) B-mNLCs group, (g) Colchicine group. Data were presented as a typical microscopic view of each group.

The severity of liver fibrosis was determined by different histological methods including H&E, Sirius Red and immunohistochemical staining. Liver sections from CCl_4_ rats demonstrated that these rats developed significant liver fibrosis (Figure 3(b–g)). Severe changes in the liver morphology were seen, including periportal and pericentral necrosis, and infiltration of inflammatory cells in the liver interstitial (Figure 3(b–g)), whereas the livers showed normal structure without any alteration (Figure 3(a)). In drug treatment groups, the liver damage was attenuated as the degree of liver fibrogenesis was decreased.

Sirius Red has been commonly used for collagen fiber staining. As shown in Figure S3, liver sections from vehicle-treated rats (positive control) displayed the most significant collagen deposition, while the background staining in Naive group was minimal. All the treatments significantly decreased the CCl_4_-induced liver collagen deposition as the intensity of Sirius Red staining in the liver sections of these rats was significantly lower than that in positive controls (*p < .*01, *n* = 9) (Figure S3h). The collagen staining in Cur-mNLCs group was the least among all these treatments, and the difference between Cur-NLCs and Cur-mNLCs was significant (*p* = .003) (Figure S3h).

The levels of Sirius Red-staining collagen fibers in the liver were further confirmed by using immunohistochemical staining of collagen I. As shown in Figure S4, the expression of collagen I was significantly induced in the livers of vehicle-treated rats as compared to the collagen I staining in naive rats. Similar to the Sirius Red staining in Figure S3, the collagen I expression in the livers was decreased by all the treatment as compared to that in positive control (*p < .*01), and the mean intensity of collagen I in Cur-mNLC group was the lowest among all these treatment groups (Figure S4h), but was not statistically different from Cur-NLCs (Figure S4h).

α-SMA is a specific marker of myofibroblasts that contribute to liver fibrosis by producing ECM including collagen (Karin et al., 2016). In this study, the anti-liver fibrosis of Cur-mNLCs compared to other treatments was further determined by the expression of α-SMA. As shown in Figure S5, the level of α-SMA expression in the liver sections from vehicle-treated positive group was significantly induced by CCl_4_ treatment, while the staining of naive rats was negative. One-way ANOVA showed that the treatment with drug preparations significantly reduced the level of α-SMA expression as compared to vehicle-treated positive group (*p < .*01) (Figure S5h), and further analysis indicated that the level of α-SMA in Cur-mNLCs group was lower than that in Cur-NLCs (*p < .*01) (Figure S5h). Taken together, all these data clearly suggested that PS-modified NLC nanoparticles were significantly more enhanced anti-fibrotic activity of Cur in the treatment of liver fibrosis.

### Treatment with Cur-mNLCs significantly up-regulated HGF expression and activated MMP-2 secretion in the liver

**Figure 4.**
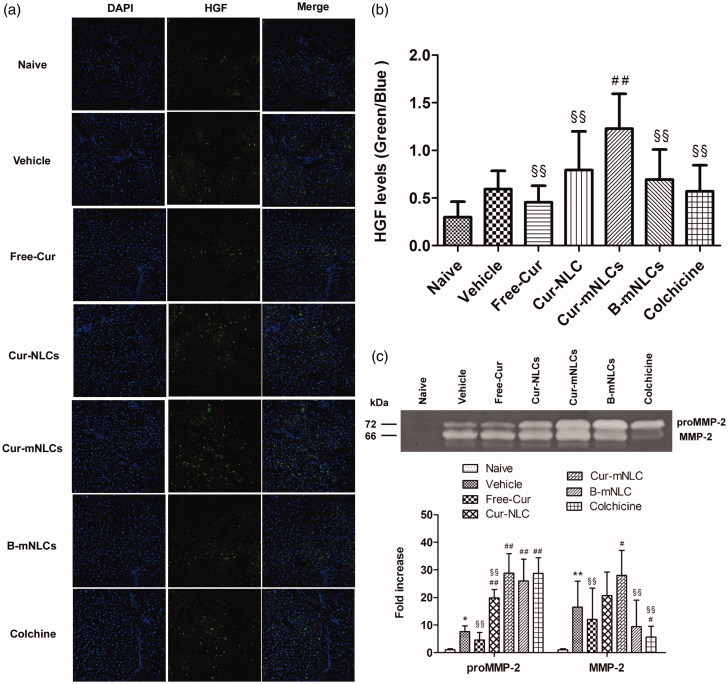
Treatment with Cur-mNLCs significantly up-regulated HGF expression and activated MMP-2 secretion in the liver. (a) Immunofluorescent staining of liver slices for antifibrotic marker HGF. (b) HGF fluorescence intensity was elevated in the livers of rats treated with different formulations in comparison with those in Vehicle group. ##*p < .*01, compared with Vehicle group, §§*p < .*01, compared with Cur-mNLCs group (mean ± SD, *n* = 6). (c) Gelatin zymography assay of proMMP-2 and MMP-2 activity showed that different drug formulations could accelerate liver fibrosis resolution by promoting MMP-2 secretion. *p < .05 or **p < .01, compared with Naive group, #*p < .*05 or ##*p < .*01, compared with Vehicle group, §§*p < .*01, compared with Cur-mNLCs group (mean ± SD, *n* = 6).

In the liver, HGF is produced by hepatocytes and promotes cell survival and tissue regeneration, and suppresses chronic inflammation and fibrosis (Nakamura et al., 2011). In this study, the correlation of HGF expression with different treatments was examined in liver sections by using immunoflurescent staining. As seen in Figure 4(a), HGF expression in the livers was significantly induced by CCl_4_ treatment as compared to the background level of Naive group. These data might indicate the initiation of tissue repair following CCl_4_-induced liver injury, and only Cur-mNLC treatment further significantly enhanced hepatic HGF level (*p < .*01) or tissue repair (Figure 4b).

Activation of MMPs secretion contributes extracellular matrix degradation that is an important feature of liver tissue repair and remodeling (Duarte et al., 2015). To further confirm the correlation of Cur-mNLCs treatment with possible HGF-mediated tissue repair or generation, the MMP-2 activation that was indicated by both proMMP-2 and MMP-2 levels was examined by using gelatin zymography assay. As shown in Figure 4(c), in contrast with almost non-detectable levels in Naive group, MMP-2 activity was significantly increased in all of CCl_4_-treated groups, and as compared to vehicle control, only the treatment with Cur-mNLCs both significantly enhanced proMMP-2 (*p < .*01) and MMP-2 (*p < .*05) activation in the liver.

## Discussion

Hepatic macrophages play an essential role in liver fibrosis, in which they participate in the initiation of inflammatory responses in injury, promotion of the fibrotic progression, but also in the regression of liver fibrosis (Affò et al., 2015). These cells can be easily targeted by nanoparticles due to their high phagocytic and endo(pino)cytic capacity (Popov et al., 2010; Ravichandran, 2011). It has been demonstrated that hepatic microenvironment might be shifted from inflammation to resolution by delivering phagocytic stimuli such as liposomes or apoptotic cells (Wynn & Barron, 2010). PS, a well-known ‘eat-me’ signal, has been widely used to decorate nanoparticles as an effective therapeutic strategy for inflammation resolution or drug/imaging agents delivery (Dvoriantchikova et al., 2009; Ogawa et al., 2014; Bagalkot et al., 2015; Ogawa et al., 2015).

There is growing interest in the clinical use of Cur as a potent anti-inflammatory agent, but the poor bioavailability of its free form due to poor absorption, rapid metabolism, and rapid systemic elimination remains unsolved (Anand et al., 2007). Our previous study has demonstrated that PS-containing carriers could enhance Cur delivery to macrophages and its anti-inflammatory activity (Wang et al., 2016). In the current study, the therapeutic effect of Cur-mNLCs on liver fibrosis was tested in a preclinical model.

Cur-mNLCs were prepared by the thin-film dispersion method previously reported (Wang et al., 2016). A conventional phosphatidylcholine NLC with Cur-loaded (Cur-NLCs) and B-mNLCs were also prepared as controls in this study. The hydrodynamic diameters of all carriers were around 200 nm, and PS decoration increased their zeta potentials. Both EE and DL of Cur in all carriers were satisfactory for nanostructured lipid carriers. Their spherical microstructures were identified by using TEM.

A rat model of liver fibrosis could be established by i.p. injection of CCl_4_ twice weekly (Shi et al., 2017). As previously reported, the clearance of nanoparticles from the peritoneal cavity depends on size and principally occurs through lymphatic duct drainage (Fu et al., 2016). Particles smaller than 50 nm are able to pass through lymph nodes while particles larger than 700 nm are mainly trapped in lymph nodes. Cur-mNLCs and Cur-NLCs (in the 50–700 nm size range) could not be transported across the peritoneum or through the lymphatics and they are likely to remain in the peritoneal cavity, and Cur drug absorption into the systemic circulation would occur only after it is released from these carriers. Then the released free drug will quickly pass through the lymphatic ducts and enter into the systemic circulation (Sigfridsson et al., 2012). Whereas the free Cur drug in Cur solution is easily diffused into the blood stream and quickly eliminated by the body. This may explain the distinct plasma drug profiles of these drug formulations.

The liver targeting properties of these drug formulations were studied by *ex-vivo* imaging and tissue distribution method. As seen in Figure 2(b), DiR-mNLCs exhibited stronger signal intensity in the livers than that of DiR-NLCs both at 2 and 4 h. Meanwhile, other organs enriched with macrophages such as the spleen and the kidney in DiR-mNLCs group also showed considerable fluorescence at 4 h. These results might be explained by the strong affinity of PS to the macrophages in these organs (Shi et al., 2007). The data of the tissue distribution revealed that Free-Cur solution was widely dispersed in most tissues and had much lower accumulation in the livers when compared with Cur-NLCs and Cur-mNLCs. This could be due to lack of targeting efficiency and passive diffusion of free Cur drug in the Cur solution. Cur-mNLCs had larger liver accumulation than Cur-NLCs, indicating PS modification improved the liver targeting ability of nanocarriers. *Ex-vivo* imaging and tissue distribution results both confirmed PS incorporation facilitated hepatic delivery of Cur-carrying nanoparticles. All of these data suggested that the mNLCs system presented in this study might improve the bioavailability of Cur, especially in the liver.

Liver fibrosis develops in response to a wide range of etiologies such as toxin exposure, viral infection, alcohol abuse, and metabolic diseases (Liu et al., 2011; Bartneck et al., 2012; Liu et al., 2012; Magdaleno et al., 2016). CCl_4_-induced liver fibrosis model was chosen in our present study as it is widely used in this field (Wu et al., 2016; Shi et al., 2017). The anti-fibrosis efficacy of different formulations in this model was examined, and the results showed that rats treated with these formulations had a remarkable reduction of liver damage in histology, reflected by reduced necrotic areas in H&E staining (Figure 3). This is also supported by the reductions in serum ALT, AST, AKP, and T-Bil, which are indicators of the liver function (Li et al., 2012; Shi et al., 2013). To assess the effects of drugs on liver fibrogenesis progression, Sirius Red staining as well as immunohistochemical staining of collagen I, the hallmark of fibrosis and α-SMA, an indicator of hepatic stellate cells (HSCs) activation (Reddy et al., 2014), were performed. As shown in Figure S3–5, the higher collagen and α-SMA staining in the livers of CCl_4_ rats were observed compared to normal rats (*p < *.01). And the therapy with drug formulations significantly reduced HSC activation and collagen deposition in chronic liver injury (*p < *.01 vs. Vehicle group).

It is well-known that deregulated inflammation is a common step of liver diseases, which would promote fibrosis progression (Seo et al., 2016). To see if Cur formulations could suppress proinflammatory cytokines associated with liver fibrosis, TNF-α, IL-1β, and IL-6 levels in serum were determined using ELISA kits. Serum collected from drug treatment rats showed significantly reduced protein levels compared with positive control group (*p < *.01).

HGF plays an important role in the inhibition of ECM accumulation and fibrogenesis in liver fibrosis, which serves as a cytoprotective, regenerative molecule as well as an antifibrotic factor. HGF protein expression is closely related to hepatic restoration (Ogaly et al., 2015; Salem et al., 2016), and can be produced mainly by Kupper cells (liver macrophages) and sinusoidal endothelial cells (Matsumoto & Nakamura, 1992). Interestingly, several studies have demonstrated that HGF expression in the macrophages is upregulated after exposure to apoptotic cells (Park et al., 2012; Byun et al., 2014). This literature evidence may imply that the up-regulated HGF in the livers of CCl_4_ rats in this study may be mainly contributed by the liver macrophages, which is further elevated by apoptotic cell-like PS-containing mNLCs. Furthermore, the up-regulated HGF elicits proliferative, survival and anti-inflammatory functions of hepatocytes (Mizuno & Nakamura, 2007), indicating that HGF is an important part of mechanism by which Cur-mNLCs reduces liver fibrosis by enhancing hepatocyte protection. This notion is supported by that when liver damages occur, considerable fluorescence was detected in fibrotic livers, indicating self-repairing of livers. And drug treatment groups showed brighter fluorescence than non-drug-treated group, which might contribute to the reduced hepatic fibrosis in these groups. However, the definitive mechanism why Cur induced HGF expression needs to be further explored. Another anti-fibrotic marker MMP-2 was also determined in this study. MMP-2 was reported to predominately influence the degree of collagen deposition and limit HSCs activities after liver injury, thereby inhibit hepatic fibrosis (Onozuka et al., 2011; Radbill et al., 2011; Giannandrea & Parks, 2014). In the current work, both pro- and active forms of MMP-2 were increased in CCl_4_ rats’ livers but not in naive rats. And drug administration further promoted MMP-2 secretion, and Cur-mNLCs exerted the most prominent effect. This result was consistent with previous reports that Cur could promote the degradation of ECM by upregulating MMP-2 expression, and inhibit connective tissue growth factor expression, TGF-β/Smad signaling and proliferation of activated HSCs, resulting in reducing liver fibrosis (Zheng & Chen, 2006; Chen et al., 2014). Taken together, these studies may indicate that Cur-mNLCs may target these fibrotic HSCs in their anti-fibrotic activities, which remains further investigation.

Collectively, our study suggested that Cur-mNLCs could be particularly useful in preventing the progression of hepatic fibrosis at multiple steps. Cur-mNLCs overcomes many defects of Cur which prevents its clinical application, and could be employed as promising drug delivery systems for liver fibrosis treatment.

## Conclusion

In the current study, we have demonstrated that administration of Cur-mNLCs could extend drug retention time *in vivo* and improve their bioavailability as shown in pharmacokinetics study. PS decoration enhanced carriers’ liver targeting properties indicated by tissue distribution as well as *ex vivo* imaging experiments. And PS exerted improved effects with Cur in reducing liver inflammation and fibrosis. The anti-fibrotic efficacies of Cur-mNLCs might be related to their hepatocytes protection, inflammation suppression, hepatocytes repairment, and collagen deposition inhibition effects.

## Supplementary Material

IDRD_Wang_et_al_Supplemental_Content.docx
