# Supplementary figures and images for: Enhanced efficacy of curcumin with phosphatidylserine-decorated nanoparticles in the treatment of hepatic fibrosis
Source: Drug Deliv. 2017 Dec 7;25(1):1–11. doi: 10.1080/10717544.2017.1399301 (PMC6058669; doi:10.1080/10717544.2017.1399301)

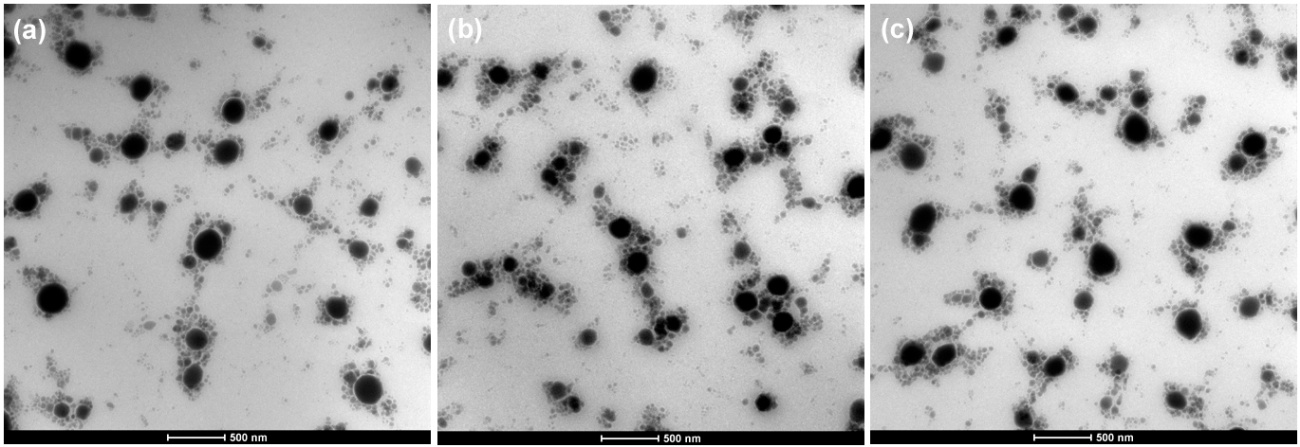


Fiugre S1


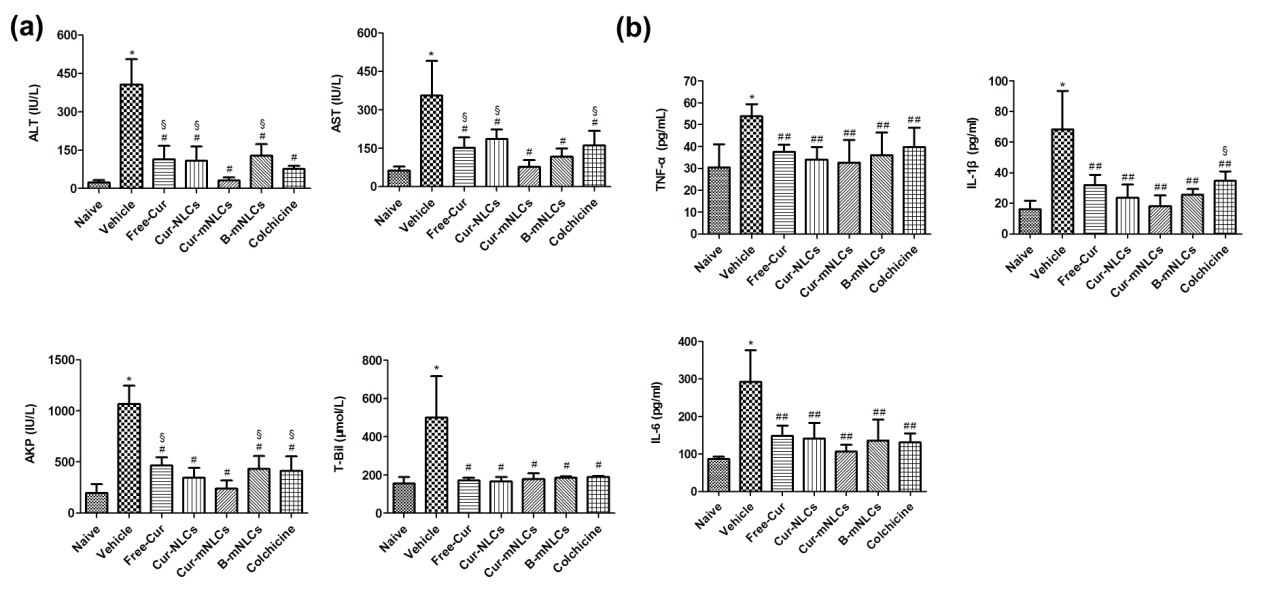


Fiugre S2


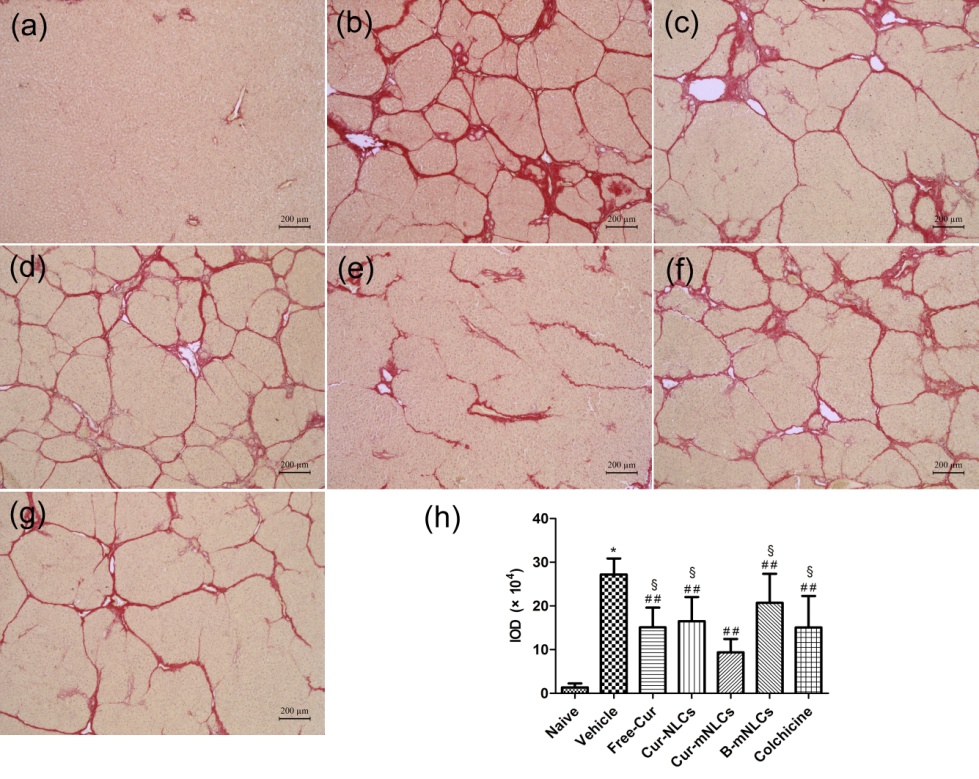


Fiugre S3


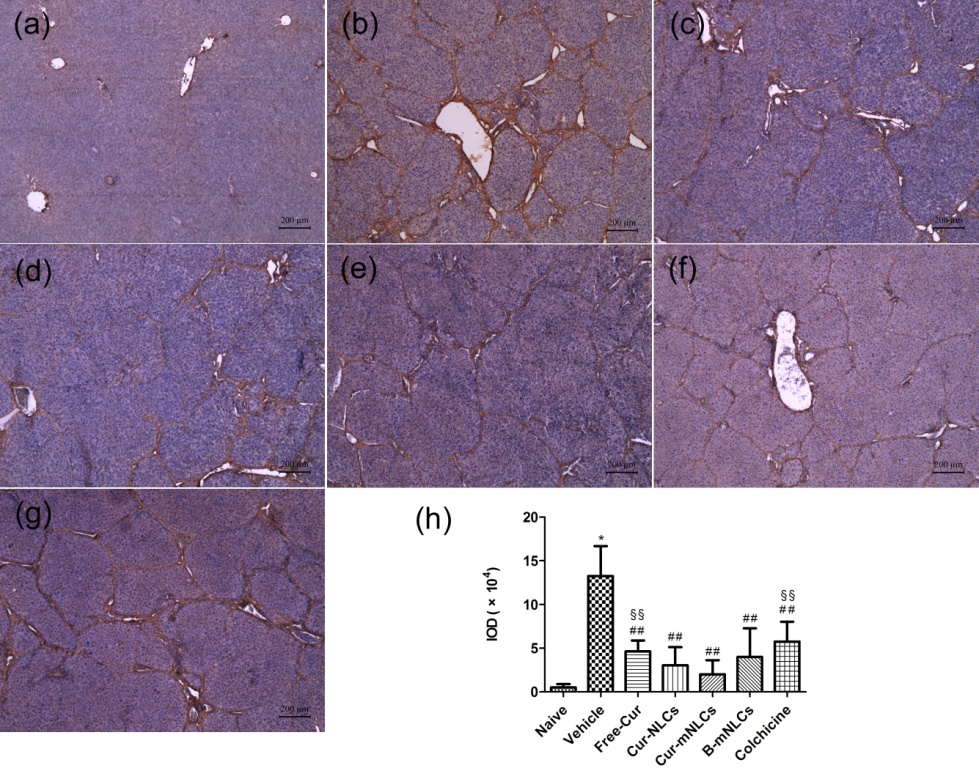


Fiugre S4


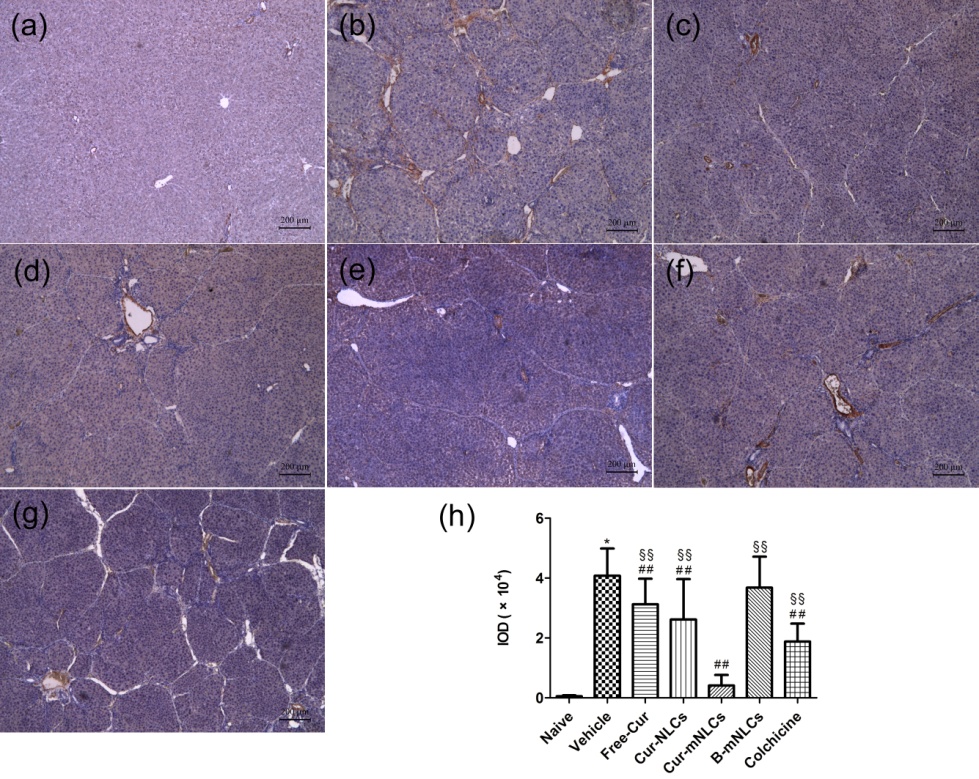


Fiugre S5

Supplement: IDRD_Wang_et_al_Supplemental_Content.docx [file IDRD_A_1399301_SM8863.docx]
